# Computational approach to grain boundary segregation engineering of nickel-base superalloys

**DOI:** 10.1038/s41598-024-63801-6

**Published:** 2024-06-06

**Authors:** Haruna Uruchida, Yuhki Tsukada, Yusuke Matsuoka, Toshiyuki Koyama

**Affiliations:** Department of Materials Design Innovation Engineering, Graduate School of Engineering, Nagoya University, Furo-Cho, Chikusa-Ku, Nagoya, 464-8603 Japan

**Keywords:** Metals and alloys, Computational methods

## Abstract

Grain boundary (GB) strengthening elements, such as B, C, and Zr have been added in small amounts to nickel-base superalloys. However, their strengthening effects have not been quantified and no specific design principles for GB chemistry have been reported. In this study, we propose a practical computational approach for the GB segregation engineering of nickel-base superalloys. Considering the partitioning of alloying elements into coexisting phases (strengthening phases, carbides, etc.), the equilibrium composition of a high-angle GB was computed for several nickel-base superalloys using a calculation of phase diagrams database. The computational results showed that B and Mo were enriched at the GB in most of the investigated alloys. The creep rupture strengths of the investigated alloys were predicted using the computed GB composition as a regression model feature. The regression coefficients for the features confirm that B segregation at the GB has a non-negligible strengthening effect on nickel-base superalloys.

## Introduction

Nickel-base superalloys exhibit significant resistance to loading under static, creep, and fatigue conditions at high temperatures and are used as materials in gas turbines for jet engines and electricity generation. Commercial nickel-base polycrystalline superalloys are designed in multicomponent and multiphase systems. The number of alloying elements used was greater than 10. The microstructure comprises γ (disordered solid solution with a face-centered cubic structure), γʹ (ordered L1_2_ compound), γʺ (ordered D0_22_ compound), and several carbide and boride types. The creep resistance of nickel-base superalloys depends on various factors, including precipitated phases (e.g., γʹ, γʺ, carbides, etc.), solid-solution strengtheners (i.e., Co, Cr, Fe, Mo, W, and Ta), diffusivity, γ phase grain size, stacking fault energy, and grain boundary (GB) strengtheners (i.e., B, C, and Zr)^1,2^. GB-strengthening elements (i.e., B, C, and Zr), which are added in small amounts to superalloys, segregate at the GB and increase creep resistance^1,2^. However, their strengthening effects have not yet been quantified; thus, no specific design principles for GB chemistry have been reported. Practical approaches for quantifying the importance of GB chemistry and regulating GB composition are required for GB segregation engineering^3^ of nickel-base superalloys.

The GB chemistry and/or microstructure of the GB region in several nickel-base superalloys have recently been experimentally investigated^4–14^. According to the literature, Cr, Mo, B, and C segregate at the GB^4–6,10–13^, and several types of carbides and borides are present near the GB region^4,6–9,11,12,14^. Accumulating experimental data on the GB chemistry of various nickel-base superalloys will shed light on its relationship with the creep resistance and lead to a deeper understanding of the role of GB-strengthening elements. Conversely, computational methods for predicting the GB chemistry are required to regulate the GB composition in multicomponent alloys. A method combining Hillert’s GB phase model^15^ and the calculation of phase diagrams (CALPHAD)^16,17^ databases was proposed^18^. This method is effective for predicting the equilibrium solute segregation to a stationary random high-angle GB. The computed GB compositions of several nickel-base superalloys, austenitic stainless steel, and a high-entropy alloy were consistent with experimental data from previous studies^18^. One of the merits of this method is that it can couple the calculation of the phase equilibrium (partitioning of alloying elements into coexisting phases) with the GB composition computation, allowing GB chemistry prediction in a multicomponent and multiphase system.

In this study, the phase equilibrium at 760 °C is computed for several nickel-base polycrystalline superalloys using a commercial CALPHAD database to collect data on the phase fractions of the coexisting phases and the γ phase composition for each alloy. The GB composition was computed for each alloy using the aforementioned method, which combines Hillert’s GB phase model and CALPHAD database^18^. First, we investigated the correlations between the computation results of the GB composition, γ phase composition, fraction of the strengthening phases, and experimental data on the 1000 h creep rupture strength at 760 °C. Second, we used the abovementioned computation results as regression model features to predict the 1000 h creep rupture strength at 760 °C. Based on the prediction performance and obtained regression coefficients for the features, we discuss the effectiveness of an approach that uses the computed GB composition to predict the creep rupture strength of nickel-base superalloys.

## Results

### Computation of phase equilibrium and GB composition

Table 1 lists the chemical compositions of several investigated solid-solution/precipitation-hardening nickel-base polycrystalline superalloys^19–27^. Some of the alloy compositional data were obtained from public data sheets (Special Metals Alloy Technical Bulletins: https://www.specialmetals.com/, Haynes International Alloy Portfolio: https://haynesintl.com/, Aubert & Duval Alloy Data Sheet: https://www.aubertduval.com/, and NIMS Creep Data Sheet: https://cds.nims.go.jp/). Note that alloys with their compositional data taken from the ASM specialty handbook^19^ were distinguished from others with an “ASM” notation (e.g., Inconel617 (ASM)). For alloys with zero or unspecified boron compositions in the literature or data sheets, a parts-per-million-level amount of boron (0.0006 wt% B) was the assumed content^20^. The phase equilibrium of each alloy at 760 °C was calculated using Thermo-Calc software^28^ and the CALPHAD database TTNI8^29^. The phases listed in Table S1 (Supplementary Material) were considered in the phase equilibrium calculation at 760 °C, which was based on literature information about the phase constituents of each alloy (reference list in Supplementary Material). Tables S2 and S3 present the calculation results of the equilibrium phase fractions and the equilibrium composition of the γ phase, respectively (Supplementary Material). The calculated composition of the γ phase was used to compute the GB chemistry using Hillert’s GB phase model^15^ and the CALPHAD database TTNI8^29^. Please refer to the “*GB composition computation*” in the “Methods” section for details. Table 2 lists the computed GB composition at 760 °C for each alloy. In Table 2 and Table S3, most of the investigated alloys showed high B and Mo concentrations at the GB when compared with those in the γ phase. Cr was also enriched in the GB of several alloys. Table 3 presents the 1000 h creep rupture strength at 760 °C for each alloy, which was taken from the ASM specialty handbook^19^, previous studies^22–27^, and public data sheets (Special Metals Alloy Technical Bulletins: https://www.specialmetals.com/; Aubert & Duval Alloy Data Sheet: https://www.aubertduval.com/; and NIMS Creep Data Sheet: https://cds.nims.go.jp/).

**Table 1:** Chemical compositions of nickel-base polycystalline superalloys investigated in this study (in wt.%)^19–27^ (DS: data sheet).

Alloy	Ni	Cr	Co	Mo	Ti	Al	W	Nb	Fe	C	B	Other	Refs.
Astroloy (ASM)	bal	15.0	15.0	5.25	3.5	4.4			0.3	0.06	0.03	0.06Zr	^19^
Hastelloy S (ASM)	bal	15.5		15.5		0.2			1.0	0.02	0.0006	0.5Mn, 0.4Si	[^19,20^,DS]
Hastelloy X (ASM)	bal	22.0	1.5	9.0			0.6		15.8	0.15	0.0006		[^19,20^,DS]
Haynes 230 (ASM)	bal	22.0	5.0	2.0		0.35	14.0		3.0	0.10	0.015	0.5Mn, 0.4Si	[^19^,DS]
Inconel 601 (ASM)	bal	23.0				1.35			14.1	0.05	0.0006	0.5Cu	^19,20^
Inconel 617 (ASM)	bal	22.0	12.5	9.0		1.0				0.07	0.002		^19,20^
Inconel 625 (ASM)	bal	21.5		9.0	0.2	0.2		3.6	2.5	0.05	0.0006		^19,20^
Inconel 718 (ASM)	bal	19.0		3.0	0.9	0.5		5.1	18.5	0.08	0.003	0.15Cu	[^19^,DS]
M-252 (ASM)	bal	19.0	10.0	10.0	2.6	1.0			0.75	0.15	0.005		^19^
Nimonic 75 (ASM)	bal	19.5			0.4	0.15			2.5	0.12	0.0006	0.25Cu	^19,20^
Nimonic 80A (ASM)	bal	19.5	1.0		2.25	1.4			1.5	0.05	0.0006	0.1Cu	^19,20^
Nimonic 90 (ASM)	bal	19.5	18.0		2.4	1.4			1.5	0.06	0.0006		^19,20^
Nimonic 105 (ASM)	bal	15.0	20.0	5.0	1.2	4.7				0.08	0.005		^19^
Nimonic 115 (ASM)	bal	15.0	15.0	4.0	4.0	5.0			1.0	0.20	0.0175	0.04Zr	[^19^,DS]
Pyromet 860 (ASM)	bal	13.0	4.0	6.0	3.0	1.0			28.9	0.05	0.01		^19^
René 41 (ASM)	bal	19.0	11.0	10.0	3.1	1.5			0.3	0.09	0.01		^19^
Udimet 500 (ASM)	bal	19.0	19.0	4.0	3.0	3.0			4.0	0.08	0.005		^19^
Udimet 520 (ASM)	bal	19.0	12.0	6.0	3.0	2.0	1.0			0.08	0.005		^19^
Udimet 700 (ASM)	bal	15.0	18.5	5.0	3.4	4.3			1.0	0.07	0.03		^19^
Udimet 710 (ASM)	bal	18.0	14.8	3.0	5.0	2.5	1.5			0.07	0.01		^19^
Unitemp AF-2 1DA6 (ASM)	bal	12.0	10.0	3.0	3.0	4.6	6.0		0.5	0.35	0.015	1.5Ta, 0.1Zr	^19^
Waspaloy (ASM)	bal	19.5	13.5	4.3	3.0	1.4			2.0	0.07	0.006	0.09Zr	^19^
AD730	bal	16.0	8.9	3.0	3.6	2.25	2.5	1.1	4.3	0.01	0.013	0.03Zr	[DS]
GTD-111	bal	13.5	9.5	1.53	4.75	3.3	3.8		0.23	0.09	0.01	2.7Ta	^22^
HR6W	bal	23.4			0.12		6.0	0.25	24.1	0.07	0.0006	1.0Mn, 0.26Si	^20,23^
Inconel 600	bal	15.5							8.0	0.075	0.0006	0.25Cu, 0.25Si	[^20^,DS]
Inconel 617	bal	22.19	14.83	9.3	0.39	1.09			1.35	0.07	0.002	0.08Mn, 0.08Si, 0.05Cu	^24^
Inconel 625	bal	21.7		9.0	0.2	0.2		3.6	2.5	0.05	0.0006		[^20^,DS]
Inconel 738LC	bal	15.81	8.3	1.76	3.47	3.46	2.61	0.97	0.08	0.1	0.01	0.04Zr, 1.72Ta, 0.01Mn	[DS]
Inconel 740	bal	24.45	19.87	0.52	1.64	1.03		1.90	0.68	0.04	0.002	0.28Mn	^25^
SINM	bal	17.5		0.5	1.8	1.6		1.2	30.0	0.03	0.005		^26^
Waspaloy	bal	19.05	14.2	4.36	2.90	1.35			0.18	0.037	0.0054		^27^

**Table 2:** Computed GB composition at 760 °C (in at.%).

Alloy	Ni	Cr	Co	Mo	Ti	Al	W	Nb	Fe	C	B	Zr
Astroloy (ASM)	bal.	28.60	16.24	15.33	0.388	0.252			0.390	0.052	9.732	0.120
Hastelloy S (ASM)	bal.	14.99		24.14		0.017			0.530	0.012	12.09	
Hastelloy X (ASM)	bal.	28.05	1.283	10.42			0.109		12.65	0.068	9.404	
Haynes 230 (ASM)	bal.	35.40	4.848	4.042		0.128	2.600		4.071	0.033	6.112	
Inconel 601 (ASM)	bal.	28.52				2.415			17.84	0.119	0.005	
Inconel 617 (ASM)	bal.	24.33	10.08	15.87		0.102				0.074	11.39	
Inconel 625 (ASM)	bal.	23.98		13.15	0.461	0.044		3.108	1.830	0.045	8.215	
Inconel718 (ASM)	bal.	24.44		5.984	1.190	0.086		3.629	17.74	0.024	7.663	
M-252 (ASM)	bal.	24.79	7.991	14.61	1.554	0.009			0.445	0.044	16.43	
Nimonic 75 (ASM)	bal.	13.29			4.995	0.559			2.259	0.539	0.114	
Nimonic 80A (ASM)	bal.	10.21	0.438		8.332	2.924			1.147	0.102	0.008	
Nimonic 90 (ASM)	bal.	11.08	10.86		7.381	3.833			1.644	0.133	0.030	
Nimonic 105 (ASM)	bal.	26.00	20.45	13.35	0.209	0.256				0.074	11.94	
Nimonic 115 (ASM)	bal.	31.80	17.99	12.03	0.386	0.493			1.929	0.058	7.585	0.099
Pyromet 860 (ASM)	bal.	15.19	3.410	13.54	4.417	0.030			22.64	0.088	14.33	
René 41 (ASM)	bal.	25.60	9.707	17.19	1.008	0.041			0.224	0.036	11.83	
Udimet 500 (ASM)	bal.	30.97	18.28	9.366	0.686	0.451			5.610	0.072	7.168	
Udimet 520 (ASM)	bal.	27.41	11.24	13.40	0.888	0.106	0.206			0.052	9.945	
Udimet 700 (ASM)	bal.	29.12	14.43	13.73	0.716	0.003			0.599	0.307	26.45	
Udimet 710 (ASM)	bal.	32.80	15.41	8.687	1.036	0.258	0.452			0.062	6.127	
Unitemp AF-2 1DA6 (ASM)	bal.	25.81	15.52	9.624	0.497	0.199	1.894		1.048	0.072	10.63	0.369
Wapaloy (ASM)	bal.	5.975	6.126	1.859	5.460	1.218			1.080	0.021	3.607	8.807
AD730	bal.	32.46	7.244	11.78	0.891	0.002	1.657	0.086	2.513	0.097	22.55	0.006
GTD-111	bal.	32.37	12.64	5.291	1.048	0.505	0.988		0.599	0.072	4.402	
HR6W	bal.	33.98			0.270		1.384	0.564	29.84	0.115	0.349	
Inconel 600	bal.	18.35							10.28	1.002	0.412	
Inconel 617	bal.	24.32	11.84	16.34	0.647	0.111			0.866	0.058	11.33	
Inconel 625	bal.	23.98		13.15	0.461	0.044		3.108	1.830	0.045	8.215	
Inconel 738LC	bal.	9.309	5.229	1.323	4.170	5.189	0.115	0.504	0.091	0.007	0.424	5.530
Inconel 740	bal.	33.50	15.61	1.649	1.442	0.063		1.160	0.784	0.064	10.49	
SINM	bal.	31.29		0.647	0.754	0.284		0.509	37.03	0.071	3.102	
Waspaloy	bal.	24.26	12.33	10.40	1.891	0.073			0.153	0.061	10.72

**Table 3.** 1000 h creep rupture strength at 760 °C^19,22–27^ (DS: data sheet).

Alloy	Creep rupture strength (MPa)	Refs.
Astroloy (ASM)	425	^19^
Hastelloy S (ASM)	90	^19^
Hastelloy X (ASM)	105	^19^
Haynes 230 (ASM)	125	^19^
Inconel 601 (ASM)	60	^19^
Inconel 617 (ASM)	165	^19^
Inconel 625 (ASM)	160	^19^
Inconel 718 (ASM)	195	^19^
M-252 (ASM)	270	^19^
Nimonic 75 (ASM)	50	^19^
Nimonic 80A (ASM)	160	^19^
Nimonic 90 (ASM)	205	^19^
Nimonic 105 (ASM)	330	^19^
Nimonic 115 (ASM)	420	^19^
Pyromet 860 (ASM)	250	^19^
René 41 (ASM)	345	^19^
Udimet 500 (ASM)	325	^19^
Udimet 520 (ASM)	345	^19^
Udimet 700 (ASM)	425	^19^
Udimet 710 (ASM)	460	^19^
Unitemp AF-2 1DA6 (ASM)	360	^19^
Waspaloy (ASM)	290	^19^
AD730	120.26	[DS]
GTD-111	422.88	^22^
HR6W	89.90	^23^
Inconel 600	38.60	[DS]
Inconel 617	139.71	^24^
Inconel 625	160.08	[DS]
Inconel 738LC	436.34	[DS]
Inconel 740	298.65	^25^
SINM	155.32	^26^
Waspaloy	315.03	^27^

### Correlation analysis

**Figure 1:**
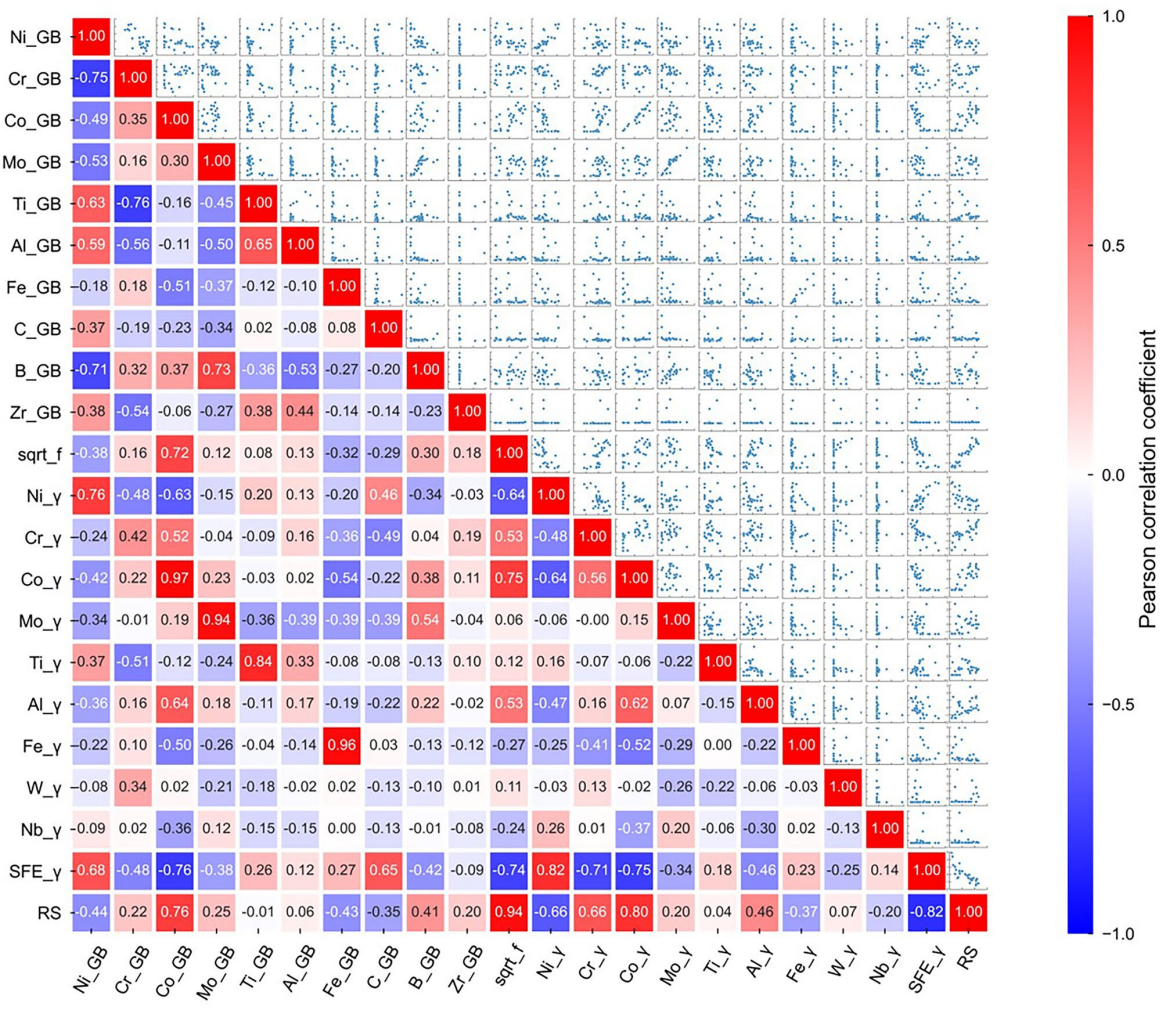
Correlation matrix and pair plots between variables: GB composition (Ni_GB, Cr_GB, Co_GB, Mo_GB, Ti_GB, Al_GB, Fe_GB, C_GB, B_GB, and Zr_GB), square root of the faction of the strengthening phases (sqrt_f), γ phase composition (Ni_γ, Cr_γ, Co_γ, Mo_γ, Ti_γ, Al_γ, Fe_γ, W_γ, and Nb_γ), stacking fault energy (SFE_γ), and 1000 h creep rupture strength at 760 °C (RS). Pearson correlation coefficients are shown in the correlation matrix.

Using the data listed in Tables 2, 3, Tables S2, and S3, we first investigated the correlations between variables, including the γ phase and GB compositions, square root of the fraction of the strengthening phases (γʹ, γʺ, and carbides were regarded as strengthening phases), stacking fault energy, and 1000 h creep rupture strength at 760 °C. The square root of the fraction of the strengthening phases was used as a variable based on the reported relationship between the creep rupture strength at 700 °C and the fraction of the strengthening phases of γʹ and γʺ in nickel-base superalloys^1^. The normalized stacking fault energy, ${{\gamma_{{{\text{SF}}}} } \mathord{\left/ {\vphantom {{\gamma_{{{\text{SF}}}} } {Gb}}} \right. \kern-0pt} {Gb}}$, was estimated from the γ phase composition ($x_{i}^{{\upgamma }}$ in atomic fraction) (Table S3).

$$\begin{aligned} \frac{{\gamma_{{{\text{SF}}}} }}{Gb} \times 10^{3} & = 12.914 - 22.4x_{{{\text{Cr}}}}^{{\upgamma}} - 11.3x_{{{\text{Co}}}}^{{\upgamma}} - 35.6x_{{{\text{Mo}}}}^{{\upgamma }} - 82.2x_{{{\text{Ti}}}}^{{\upgamma }} \\ & \quad - 23.8x_{{{\text{Al}}}}^{{\upgamma}} - 8.26x_{{{\text{Fe}}}}^{{\upgamma}} - 72.0x_{{\text{W}}}^{{\upgamma}} - 27.1x_{{{\text{Nb}}}}^{{\upgamma}} \\ \end{aligned}$$

here, *G* is the shear modulus, and *b* is the Burgers vector magnitude^30^. Figure 1 presents the correlation matrix and pair plots. Pearson correlation coefficients (PCCs) were depicted in the correlation matrix. A large positive PCC value indicates a strong positive correlation between two variables, whereas a large negative PCC value indicates a strong negative correlation between two variables. The Co, Mo, Ti, and Fe concentrations at the GB exhibited strong positive correlations with those in the γ phase, indicating that the concentrations of these solute elements at the GB increased as their γ phase concentrations increased. A positive correlation was observed between the Mo and B concentrations at the GB, implying that Mo and B tended to co-segregate at the GB, as confirmed by the computed GB compositions of the investigated alloys (Table 2). In contrast, no clear correlation was observed between Cr and B concentrations at the GB. The creep rupture strength showed a strong positive correlation with the square root of the fraction of the strengthening phases (sqrt_f) and a strong negative correlation with the stacking fault energy (SFE_γ), which was reasonable because the creep resistance increased as the amount of the strengthening phases increased and as the stacking fault energy decreased^1,2,31^. The rupture strength was also positively correlated with the Cr and Co concentrations in the γ phase. Cr and Co are solid-solution strengthening elements that also contributes to the reduction of the stacking fault energy^1,2,32^. Clear correlations cannot be observed between the rupture strength and the B, C, and Zr concentrations at the GB, which are GB-strengthening elements^1,2^.

### Prediction of creep rupture strength

**Figure 2:**
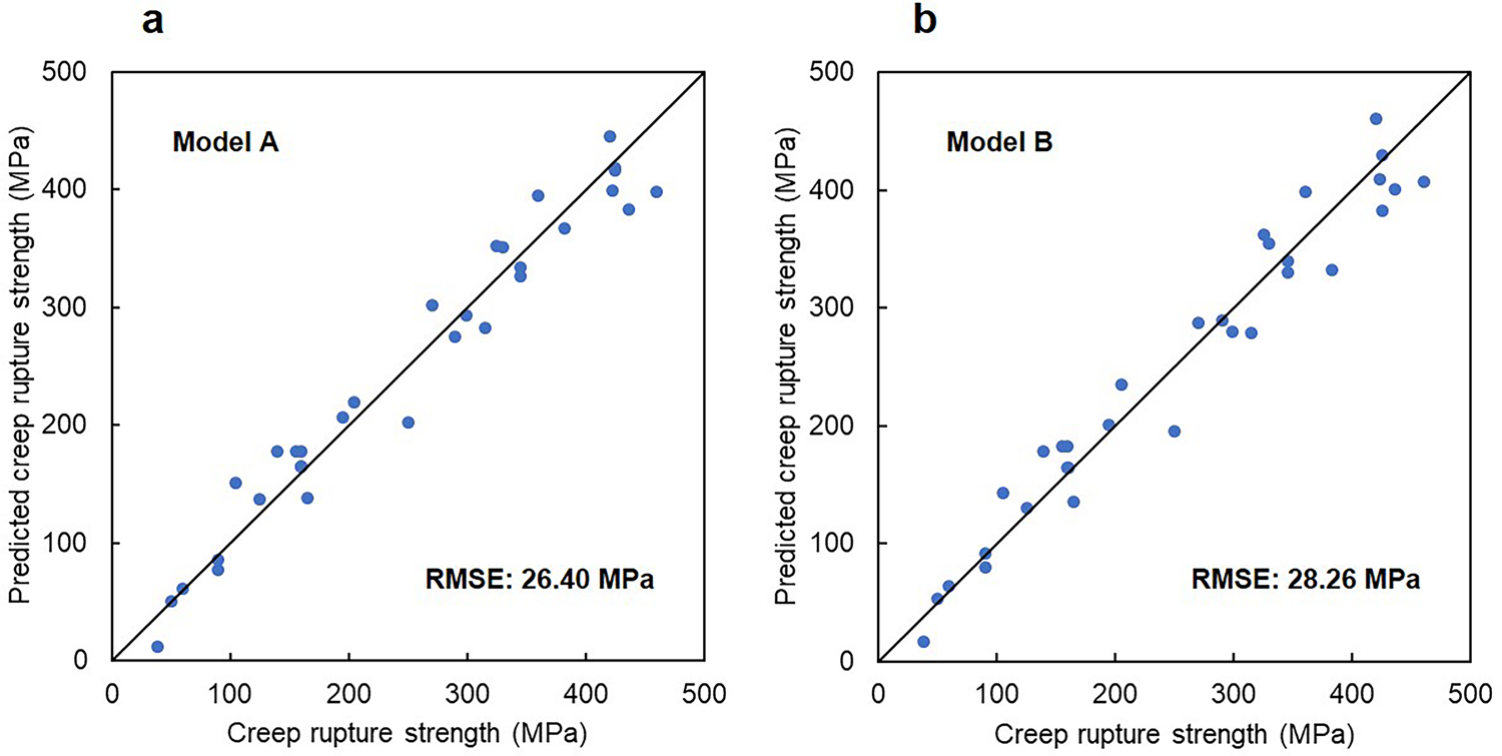
Prediction performance of the regression models. The horizontal axis represents the true creep rupture strength. The vertical axis represents the predicted creep rupture strength. (**a**) Result for the regression model, including the GB chemistry in the features (Model A). (**b**) Result for the regression model, excluding the GB chemistry from the features (Model B).

Next, we aimed to predict the creep resistance of nickel-base polycrystalline superalloys using regression analysis. Let the target property be a function of the feature. The 1000 h creep rupture strength at 760 °C (Table 3) was used as the target property. The γ phase (Table S3) and GB compositions (Table 2) and the square root of the fraction of the strengthening phases (Table S2) were used as the regression model features. The grain size of the γ phase was not included in the feature because the grain sizes in the investigated alloys were not available in the literature. Because the collected data were small, LASSO was used for feature selection. Please refer to the “Regression analysis using linear model” in the “Methods” section for details. Figure 2a demonstrates the prediction performance when GB chemistry was included in the features (Model A). The horizontal axis represents the true creep rupture strength, and the vertical axis represents the predicted creep rupture strength. We see that the creep rupture strength was successfully predicted. The prediction error (root mean square error (RMSE)) was 26.40 MPa. Conversely, Fig. 2b illustrates the prediction performance when GB chemistry was excluded from the features (Model B). The prediction error (RMSE) was 28.26 MPa, which was slightly reduced by including the GB composition in the regression model features.

**Figure 3:**
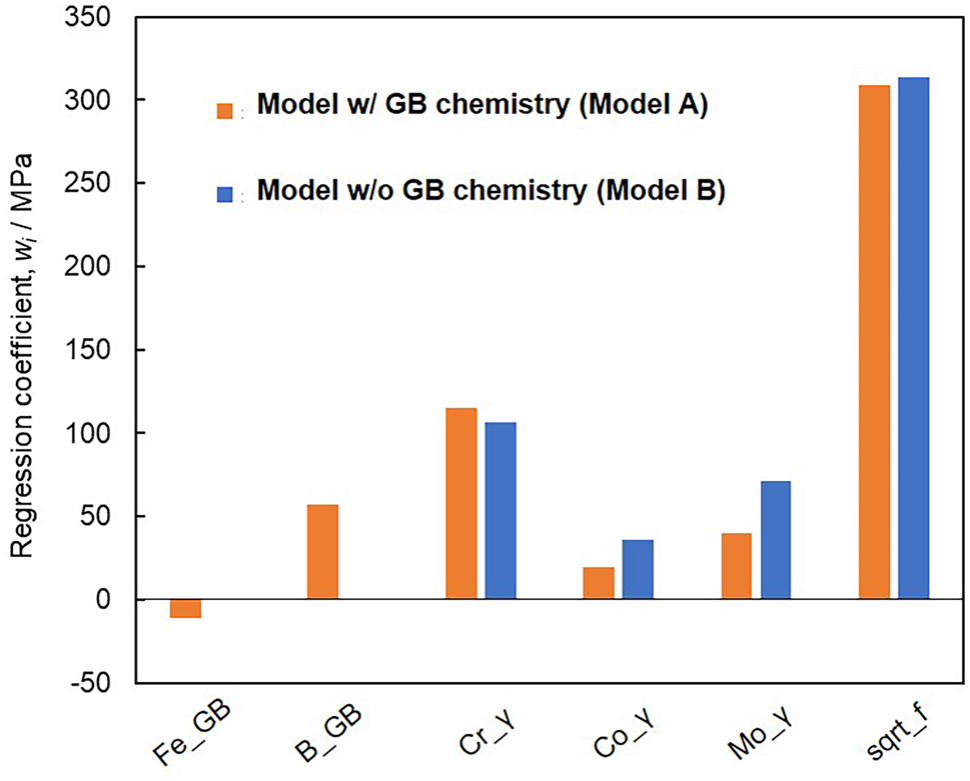
Regression coefficient values for the features selected by LASSO. The Fe and B concentrations at GB (i.e., Fe_GB and B_GB), Cr, Co, and Mo concentrations in the γ phase (i.e., Cr_γ, Co_γ, and Mo_γ), and square root of the fraction of the strengthening phases (sqrt_f) are selected as features. The result in orange represents the regression model including the GB chemistry in the features (Model A), while that in blue depicts the regression model excluding the GB chemistry from the features (Model B).

Figure 3 shows the regression coefficients for the LASSO-selected features. The result in orange is for “Model A,” while that in blue is for “Model B.” The square root of the fraction of the strengthening phases (sqrt_f) and the Cr, Co, and Mo concentrations in the γ phase were selected as features for both models. The Fe and B concentrations at the GB were selected as the features of Model A. The regression coefficient for sqrt_f exhibited the largest positive value among the selected features, which is reasonable because the creep resistance of nickel-base superalloys is significantly increased by increasing the amount of strengthening phases^1,2^. The selected features of the Cr, Co, and Mo concentrations in the γ phase, which obtained positive regression coefficients, were effective in increasing the creep resistance. These elements are solid-solution strengtheners that also lower stacking fault energy^1,2,32^. More importantly, the B concentration at the GB exhibited a positive regression coefficient (Model A) and significantly contributed to the increase in the creep rupture strength. The Fe concentration at the GB displayed a small negative regression coefficient (Model A), which remains an open question. The reason for selecting Fe concentration at the GB as a feature should be clarified in future studies.

## Discussion

The GB of nickel-base superalloys exposed to high temperatures was assumed to have a nearly equilibrium GB phase composition. Regression analysis using a linear model showed that the computed equilibrium GB composition was useful as a feature for predicting creep rupture strength. The B concentration at the GB had a non-negligible effect on the creep resistance of the nickel-base superalloys. Note that although the prediction error of the creep rupture strength does not significantly differ between Models A and B (Fig. 2), Model A, which includes GB chemistry in its features, should gain broad acceptance because there has been experimental evidence that B segregates at GBs^5,6,11,13^, which can improve GB cohesion and strength^33^. In addition, note that the GB composition was directly used as a feature in model A because the detailed/dominant role of B segregation on the GB-strengthening and creep resistance of nickel-base superalloys is controversial^14^; B segregation would change the GB properties by improving the GB cohesion^33^ and can also change the GB microstructure by inducing boride formation, which may result in GB serrations and improve ductility^8^. If a physical model that links B segregation at the GB to creep resistance is established in the future, physics-based features can be defined using the GB composition, which might allow creep rupture strength prediction with higher accuracy.

In the GB composition computation, the phase equilibrium in a multiphase and multicomponent system was first calculated to obtain the equilibrium composition of the γ phase. The GB composition strongly depended on the phase constituent because the partitioning of the alloying elements into coexisting phases (e.g., γʹ, γʺ, carbides, etc.) lowered the concentrations of the alloying elements in the γ phase, consequently decreasing the concentrations of those at the GB. In this study, the constituent phases considered in the calculations (Table S1) were determined from the literature on the microstructure of each alloy. Notably, phase transitions can occur in the nickel-base superalloys during long-term use at high temperatures. For example, a common carbide reaction sequence in nickel-base superalloys is from MC to M_23_C_6_, where M represents metallic elements^1,2,9^. However, considering these phase transitions when computing the GB chemistry is difficult, as in the Larson–Miller plots of high-temperature creep data^34^.

The GB chemistry computations revealed that B and Mo were enriched at the GB in most of the investigated alloys, whereas Cr was enriched at the GB in several alloys. Correlation analysis confirmed a positive correlation between the Mo and B concentrations at the GB, indicating that Mo was not only useful for solid-solution strengthening and stacking fault energy reduction but also played an important role in B segregation at the GB. The B, Cr, and/or Mo segregation at the GB may induce boride formation (e.g., Cr-rich M_5_B_3_^7–9^, Cr- and/or Mo-rich M_2_B^4,14^, or M_5_B_3_^11^). The presence of borides at the GB and their effects on the changes in the microstructure (morphology and composition of the constituent phases) near the GB region were intensively investigated to reveal the relationship between the microstructure and creep damage at the GB^7–9^. Although the effect of borides on the creep resistance of nickel-base superalloys is not fully understood, the computation of GB chemistry should play a central role in regulating both the GB composition and segregation-induced precipitation of borides at the GB.

Zr is a GB-strengthening element, whereas W is a solid-solution strengthening one^1,2,35^. However, the Zr concentration at the GB and the W concentration in the γ phase were not selected as features for the creep rupture strength prediction because only a few alloys among the investigated ones contained W and/or Zr (refer to Table 1). This obscures the strengthening effect of the alloying elements. C is a GB-strengthening element^1,2^, but it primarily exists in several carbide types, which are considered to be constituent phases in the phase equilibrium calculation at 760 °C (Table S1). This seems to be why the C concentration at the GB was not selected as a feature for the creep rupture strength prediction. The grain size of the γ phase not considered as a model feature herein is one of the major factors affecting the creep behavior of the nickel-base polycrystalline superalloys^1,2^. Although the grain sizes in the investigated commercial alloys were not available in the literature, they were assumed to be optimized below 100 μm to achieve the combination of tensile, creep, and fatigue properties^1,2,36,37^. In future studies, information on the grain sizes of the γ phase in various nickel-base superalloys should be collected and used as a regression model feature, which would allow the strengthening effect quantification and the creep rupture strength prediction with significantly higher accuracy.

In this study, we demonstrated that a regression model, wherein the computation results (i.e., the fraction of the strengthening phases and the γ and GB phase compositions) were used as features, can successfully predict the creep rupture strength of the nickel-base polycrystalline superalloys. We also showed that B segregation at the GB significantly contributes to an increase in the creep resistance of nickel-base superalloys. The computational approach proposed herein is applicable to nickel-base superalloys fabricated via powder metallurgy processes^1,2^ and various heat-resistant steels. Material developers/manufacturers are assumed to own large non-public databases on microstructures (e.g., grain size, constituent phases, etc.), microstructure evolution during high-temperature exposure, and mechanical properties for various heat-resistant alloys. The combination of these existing databases with the proposed computational approach opens a promising pathway to GB segregation engineering of multicomponent and multiphase heat-resistant alloys.

## Methods

### GB composition computation

**Figure 4:**
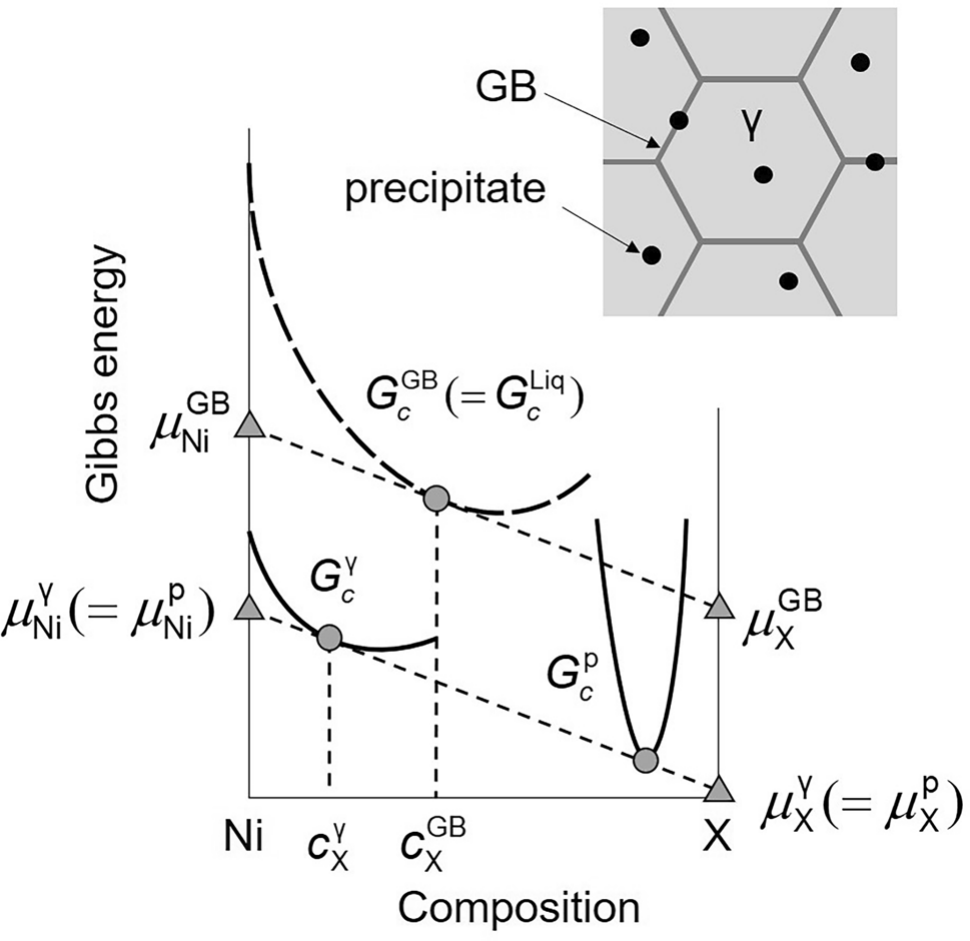
Gibbs energy–composition diagram showing the concept of parallel-tangent law in a Ni–X binary system. When the γ phase is in equilibrium with a precipitate phase, the phase equilibrium is first calculated to obtain the composition of the γ phase ($c_{{\text{X}}}^{{\upgamma }}$). Then, the composition of the GB phase ($c_{{\text{X}}}^{{{\text{GB}}}}$) can be found by a parallel-tangent construction to Gibbs energy curves of the γ and GB phases. Note that the Gibbs energy of the liquid phase is assigned to that of the GB phase^18^.

The GB region is regarded as a homogeneous-phase thin film with constant thickness^15^. We considered minimizing the Gibbs energy of a grain phase (γ) and GB phase mixture under a fixed GB phase fraction in an *N*-component system. The equilibrium composition of the GB phase at constant temperature was determined from the following relationship, assuming that the GB phase fraction was infinitesimally small:

$$\frac{{\partial G_{c}^{{\upgamma }} }}{{\partial {\mathbf{c}}_{{\upgamma }} }} = \frac{{\partial G_{c}^{{{\text{GB}}}} }}{{\partial {\mathbf{c}}_{{{\text{GB}}}} }}$$

here, $G_{c}^{{\upgamma }}$ and $G_{c}^{{{\text{GB}}}}$ are the Gibbs energies of the γ and GB phases, respectively, and ${\mathbf{c}}_{{\upgamma }} = (c_{1}^{{\upgamma }} ,c_{2}^{{\upgamma }} , \ldots ,c_{N - 1}^{{\upgamma }} )^{{\text{T}}}$ and ${\mathbf{c}}_{{{\text{GB}}}} = (c_{1}^{{{\text{GB}}}} ,c_{2}^{{{\text{GB}}}} , \ldots ,c_{N - 1}^{{{\text{GB}}}} )^{{\text{T}}}$ are the composition vectors of the γ and GB phases, respectively^18^. $\, \cdot \,^{{\text{T}}}$ represents the $\, \cdot \,$ transpose. The Nelder–Mead method^38^ was used to compute the optimum ${\mathbf{c}}_{{{\text{GB}}}}$ that minimized the following penalty function:

$$\begin{aligned} J & = \left( {\frac{{\partial G_{c}^{{\upgamma }} }}{{\partial {\mathbf{c}}_{{\upgamma }} }} - \frac{{\partial G_{c}^{{{\text{GB}}}} }}{{\partial {\mathbf{c}}_{{{\text{GB}}}} }}} \right)^{2} \\ & = \left\{ {\left( {{{\varvec{\upmu}}}_{{\upgamma }} - {{\varvec{\upmu}}}_{{{\text{GB}}}} } \right) - {\mathbf{e}}\left( {\mu_{N}^{{\upgamma }} - \mu_{N}^{{{\text{GB}}}} } \right)} \right\}^{2} \\ \end{aligned}$$

here, ${\mathbf{e}} = (1,1, \ldots ,1)^{{\text{T}}}$, and ${{\varvec{\upmu}}}_{{\upgamma }} = (\mu_{1}^{{\upgamma }} ,\mu_{2}^{{\upgamma }} , \ldots ,\mu_{N - 1}^{{\upgamma }} )^{{\text{T}}}$ and ${{\varvec{\upmu}}}_{{{\text{GB}}}} = (\mu_{1}^{{{\text{GB}}}} ,\mu_{2}^{{{\text{GB}}}} , \ldots ,\mu_{N - 1}^{{{\text{GB}}}} )^{{\text{T}}}$ denote the chemical potential vectors of the γ and GB phases, respectively. The Gibbs energy of the liquid phase was assigned to that of the GB phase, which was confirmed to be effective in predicting the equilibrium composition of random high-angle GB in nickel-base superalloys, austenitic stainless steel, and a high-entropy alloy^18^. The chemical potentials were computed using Thermo-Calc software^28^ and the CALPHAD database TTNI8^29^. TC-Python was employed to couple the Thermo-Calc computations with an in-house Python code. Figure 4 illustrates the concept of Hillert’s GB phase model (parallel-tangent law) for computing GB phase composition. In a multiphase system, the phase equilibrium was first calculated using the Thermo-Calc software and TTNI8 to obtain ${\mathbf{c}}_{{\upgamma }}$. This was followed by the computation of ${\mathbf{c}}_{{{\text{GB}}}}$ using in-house Python code. The optimum ${\mathbf{c}}_{{{\text{GB}}}}$ satisfied *J* < 1 J^2^ mol^−2^.

### Regression analysis using linear model

Regression analysis was performed using scikit-learn^39^. The loss function for the LASSO regression is given as:

$$L_{{{\text{LASSO}}}} = \sum\limits_{k = 1}^{N} {\left( {w_{0} + \sum\limits_{j = 1}^{n} {w_{j} X_{j}^{(k)} } - Y^{(k)} } \right)^{2} + \alpha \sum\limits_{j = 1}^{n} {\left| {w_{j} } \right|} }$$

here, $Y$ is the target property, $X_{j}$ is the feature, $w_{0}$ is the intercept, $w_{j}$ is the regression coefficient, $\alpha$ is the regularization parameter, $N$ is the number of investigated alloys, and $n$ is the number of features. Each feature is normalized using its minimum and maximum values. $\alpha$ was determined through leave-one-out cross-validation to maximize model generalizability. The features of $X_{j}$ with the regression coefficients of $\left| {w_{j} } \right| < 1$ were manually excluded.

## Supplementary Information

Supplementary Information.

## Data Availability

The authors declare that the data supporting the findings of this study are available within the manuscript or supplementary material.
