# Supplementary material for: Computational approach to grain boundary segregation engineering of nickel-base superalloys
Source: Sci Rep. 2024 Jun 6;14:12996. doi: 10.1038/s41598-024-63801-6 (PMC11637035; doi:10.1038/s41598-024-63801-6)
Supplement: Supplementary file 1 — Supplementary Information. [file 41598_2024_63801_MOESM1_ESM.pdf]

# Supplementary Material

Computational approach to grain boundary segregation engineering of nickel-base superalloys

Haruna Uruchida, Yuhki Tsukada, Yusuke Matsuoka, Toshiyuki Koyama

Table S1 Phase constituents considered in the equilibrium calculation at 760 °C [1–22]. In the CALPHAD method, MC carbide was modeled as a C-rich γ phase; thus, it was inevitably considered in the equilibrium calculation (DS: public data sheet).

| Alloy | Phase | Refs. |
|---|---|---|
| Astroloy (ASM) | γ, γ′, $M_{23}C_6$, MC | [1,2] |
| Hastelloy S (ASM) | γ, $M_{23}C_6$, MC, $M_6C$ | |
| Hastelloy X (ASM) | γ, $M_{23}C_6$, MC, $M_6C$, σ | [3,4] |
| Haynes 230 (ASM) | γ, $M_{23}C_6$, MC, $M_6C$ | [5−7] |
| Inconel 601 (ASM) | γ, $M_{23}C_6$, MC, $M_6C$ | |
| Inconel 617 (ASM) | γ, γ′, $M_{23}C_6$, MC | [8] |
| Inconel 625 (ASM) | γ, γ″, $M_{23}C_6$, MC, $M_6C$ | [DS] |
| Inconel 718 (ASM) | γ, γ′, γ″, $M_{23}C_6$, MC | [9] |
| M-252 (ASM) | γ, γ′, MC, $M_6C$, μ | [9] |
| Nimonic 75 (ASM) | γ, $M_{23}C_6$, MC, $M_7C_3$ | [9] |
| Nimonic 80A (ASM) | γ, γ′, $M_{23}C_6$, MC, $M_7C_3$ | [9] |
| Nimonic 90 (ASM) | γ, γ′, $M_{23}C_6$, MC | [9] |
| Nimonic 105 (ASM) | γ, γ′, $M_{23}C_6$, MC | [10] |
| Nimonic 115 (ASM) | γ, γ′, $M_{23}C_6$, MC | |
| Pyromet 860 (ASM) | γ, γ′, $M_{23}C_6$, MC | |
| René 41 (ASM) | γ, γ′, $M_{23}C_6$, MC, $M_6C$ | [9] |
| Udimet 500 (ASM) | γ, γ′, $M_{23}C_6$, MC | [9] |
| Udimet 520 (ASM) | γ, γ′, $M_{23}C_6$, MC | [11,12] |
| Udimet 700 (ASM) | γ, γ′, MC, σ | [13] |
| Udimet 710 (ASM) | γ, γ′, $M_{23}C_6$, MC | |
| Unitemp AF-2 1DA6 (ASM) | γ, γ′, $M_{23}C_6$, MC | |
| Waspaloy (ASM) | γ, γ′, $M_{23}C_6$, MC, $M_6C$, Laves | [9] |
| AD730 | γ, γ′, MC | [14] |
| GTD-111 | γ, γ′, $M_{23}C_6$, MC, η, Laves, δ, σ | [15,16] |
| HR6W | γ, $M_{23}C_6$, MC | [17,18] |
| Inconel 600 | γ, $M_{23}C_6$, MC, $M_7C_3$ | [DS] |
| Inconel 617 | γ, γ′, $M_{23}C_6$, MC | [8] |
| Inconel 625 | γ, γ″, $M_{23}C_6$, MC, $M_6C$ | [DS] |
| Inconel 738LC | γ, γ′, $M_{23}C_6$, MC | [19] |
| Inconel 740 | γ, γ′, MC | [20] |
| SINM | γ, γ′, $M_{23}C_6$, MC | [21] |
| Waspaloy | γ, γ′, $M_{23}C_6$, MC | [22] |

Table S2 Calculated equilibrium phase fractions at 760 °C.

| Alloys | $\gamma$ (#1) | $\gamma$ (#2) | $\gamma$ (#3) | $\gamma'$ | $\gamma''$ | $M_{23}C_6$ (#1) | $M_{23}C_6$ (#2) | $M_{23}C_6$ (#3) | $M_6C$ | $M_7C_3$ | $\sigma$ | $\mu$ |
|---|---|---|---|---|---|---|---|---|---|---|---|---|
| Astroloy (ASM) | 0.4553 | | | 0.5241 | | 0.0203 | 0.0003 | | | | | |
| Hastelloy S (ASM) | 0.9930 | | | | | | | | 0.0070 | | | |
| Hastelloy X (ASM) | 0.9178 | | | | | 0.0006 | 0.0003 | | 0.0498 | | 0.0315 | |
| Haynes 230 (ASM) | 0.9631 | | | | | 0.0121 | | | 0.0248 | | | |
| Inconel 601 (ASM) | 0.9887 | | | | | 0.0113 | | | | | | |
| Inconel 617 (ASM) | 0.9835 | | | | | 0.0083 | 0.0083 | | | | | |
| Inconel 625 (ASM) | 0.9471 | | | | 0.0388 | 0.0002 | 0.0071 | | 0.0068 | | | |
| Inconel 718 (ASM) | 0.8401 | 0.0079 | | 0.0701 | 0.0802 | 0.0017 | | | | | | |
| M-252 (ASM) | 0.7437 | | | 0.1852 | | | | | 0.0507 | | | 0.0204 |
| Nimonic 75 (ASM) | 0.9818 | | | | | | | | | 0.0182 | | |
| Nimonic 80A (ASM) | 0.8391 | | | 0.1497 | | 0.0112 | | | | | | |
| Nimonic 90 (ASM) | 0.8069 | | | 0.1798 | | 0.0134 | | | | | | |
| Nimonic 105 (ASM) | 0.5692 | | | 0.4120 | | 0.0167 | | 0.0021 | | | | |
| Nimonic 115 (ASM) | 0.3551 | | | 0.5968 | | 0.0360 | 0.0121 | | | | | |
| Pyromet 860 (ASM) | 0.8030 | | | 0.1833 | | 0.0057 | 0.0081 | | | | | |
| René 41 (ASM) | 0.7113 | | | 0.2577 | | | 0.0057 | | 0.0253 | | | |

Table S2 *continued*.

| Alloys | γ (#1) | γ (#2) | γ (#3) | γ′ | γ″ | $M_{23}C_6$ (#1) | $M_{23}C_6$ (#2) | $M_{23}C_6$ (#3) | $M_6C$ | $M_7C_3$ | σ | μ |
|---|---|---|---|---|---|---|---|---|---|---|---|---|
| Udimet 500 (ASM) | 0.6315 | | | 0.3495 | | 0.0158 | 0.0032 | | | | | |
| Udimet 520 (ASM) | 0.6909 | | | 0.2896 | | 0.0168 | 0.0026 | | | | | |
| Udimet 700 (ASM) | 0.3844 | 0.0063 | | 0.5034 | | | | | | | 0.1058 | |
| Udimet 710 (ASM) | 0.5345 | | | 0.4473 | | 0.0078 | 0.0104 | | | | | |
| Unitemp AF-2 1DA6 (ASM) | 0.3022 | | | 0.6136 | | 0.0754 | 0.0087 | | | | | |
| Waspaloy (ASM) | 0.7496 | 0.0002 | | 0.2335 | | 0.0034 | 0.0133 | | | | | |
| AD730 | 0.6228 | 0.0009 | | 0.3763 | | | | | | | | |
| GTD-111 | 0.3974 | | | 0.5720 | | 0.0142 | 0.0091 | | | | 0.0073 | |
| HR6W | 0.9845 | | 0.0008 | | | 0.0142 | 0.0005 | | | | | |
| Inconel 600 | 0.989 | | | | | | | | | 0.0110 | | |
| Inconel 617 | 0.9578 | | | 0.0257 | | 0.0165 | | | | | | |
| Inconel 625 | 0.9471 | | | | 0.0388 | 0.0002 | 0.0071 | | 0.0068 | | | |
| Inconel 738LC | 0.4598 | | | 0.5149 | | 0.0092 | 0.0161 | | | | | |
| Inconel 740 | 0.8217 | 0.0036 | | 0.1747 | | | | | | | | |
| SINM | 0.8230 | 0.0018 | | 0.1714 | | 0.0039 | | | | | | |
| Waspaloy | 0.7694 | | | 0.2213 | | 0.0068 | | 0.0025 | | | | |

Table S3 Calculated equilibrium composition of γ phase at 760 °C (in at.%).

| Alloy | Ni | Cr | Co | Mo | Ti | Al | W | Nb | Fe | C | B | Zr |
|---|---|---|---|---|---|---|---|---|---|---|---|---|
| Astroloy (ASM) | bal. | 28.60 | 21.54 | 5.752 | 0.101 | 2.642 | | | 0.554 | 0.001 | 0.005 | 0.004 |
| Hastelloy S (ASM) | bal. | 18.12 | | 9.541 | | 0.453 | | | 1.089 | <0.001 | 0.006 | |
| Hastelloy X (ASM) | bal. | 24.56 | 1.547 | 3.199 | | | 0.140 | | 17.23 | 0.002 | 0.003 | |
| Haynes 230 (ASM) | bal. | 26.06 | 5.454 | 1.199 | | 0.841 | 4.039 | | 3.439 | 0.001 | 0.001 | |
| Inconel 601 (ASM) | bal. | 24.03 | | | | 2.817 | | | 14.19 | 0.002 | <0.001 | |
| Inconel 617 (ASM) | bal. | 24.07 | 12.51 | 5.386 | | 2.195 | | | | 0.002 | 0.007 | |
| Inconel 625 (ASM) | bal. | 25.34 | | 5.483 | 0.207 | 0.457 | | 1.550 | 2.801 | 0.002 | 0.003 | |
| Inconel718 (ASM) | bal. | 24.86 | | 2.091 | 0.242 | 0.646 | | 0.601 | 22.48 | 0.001 | 0.002 | |
| M-252 (ASM) | bal. | 26.17 | 11.54 | 4.765 | 0.572 | 0.801 | | | 0.995 | 0.003 | 0.036 | |
| Nimonic 75 (ASM) | bal. | 20.40 | | | 0.483 | 0.322 | | | 2.587 | 0.027 | <0.001 | |
| Nimonic 80A (ASM) | bal. | 23.53 | 1.068 | | 1.101 | 1.608 | | | 1.757 | 0.008 | <0.001 | |
| Nimonic 90 (ASM) | bal. | 24.38 | 19.60 | | 0.612 | 1.376 | | | 1.822 | 0.010 | <0.001 | |
| Nimonic 105 (ASM) | bal. | 23.36 | 25.67 | 3.699 | 0.063 | 4.155 | | | | 0.001 | 0.007 | |
| Nimonic 115 (ASM) | bal. | 30.48 | 23.40 | 4.379 | 0.087 | 2.706 | | | 2.169 | 0.001 | 0.003 | 0.002 |
| Pyromet 860 (ASM) | bal. | 16.53 | 4.373 | 4.305 | 0.572 | 0.944 | | | 35.50 | 0.003 | 0.004 | |
| René 41 (ASM) | bal. | 27.91 | 13.36 | 7.010 | 0.321 | 1.020 | | | 0.412 | 0.002 | 0.010 | |

Table S3 *continued.*

| Alloy | Ni | Cr | Co | Mo | Ti | Al | W | Nb | Fe | C | B | Zr |
|---|---|---|---|---|---|---|---|---|---|---|---|---|
| Udimet 500 (ASM) | bal. | 29.03 | 23.23 | 3.261 | 0.142 | 2.233 | | | 5.867 | 0.001 | 0.003 | |
| Udimet 520 (ASM) | bal. | 27.58 | 14.66 | 4.818 | 0.291 | 1.466 | 0.367 | | | 0.002 | 0.005 | |
| Udimet 700 (ASM) | bal. | 25.22 | 25.47 | 3.025 | 0.130 | 2.884 | | | 1.936 | 0.030 | 0.398 | |
| Udimet 710 (ASM) | bal. | 32.42 | 20.19 | 3.000 | 0.223 | 1.072 | 0.609 | | | 0.002 | 0.002 | |
| Unitemp AF-2 1DA6 (ASM) | bal. | 21.88 | 18.31 | 2.649 | 0.181 | 2.666 | 2.648 | | 1.283 | 0.002 | 0.004 | 0.013 |
| Waspaloy (ASM) | bal. | 26.45 | 15.58 | 3.173 | 0.457 | 1.027 | | | 2.622 | 0.002 | 0.006 | 0.018 |
| AD730 | bal. | 27.34 | 11.67 | 2.795 | 0.287 | 1.260 | 0.967 | 0.047 | 6.627 | 0.010 | 0.111 | 0.002 |
| GTD-111 | bal. | 29.66 | 15.73 | 1.740 | 0.260 | 1.322 | 1.791 | | 0.505 | 0.002 | 0.001 | |
| HR6W | bal. | 25.60 | | | 0.143 | | 1.839 | 0.119 | 25.50 | 0.002 | <0.001 | |
| Inconel 600 | bal. | 16.41 | | | | | | | 8.251 | 0.031 | <0.001 | |
| Inconel 617 | bal. | 24.78 | 15.07 | 5.693 | 0.230 | 2.116 | | | 1.459 | 0.002 | 0.006 | |
| Inconel 625 | bal. | 25.34 | | 5.483 | 0.207 | 0.457 | | 1.550 | 2.801 | 0.002 | 0.003 | |
| Inconel 738LC | bal. | 31.24 | 13.01 | 1.807 | 0.179 | 1.688 | 1.051 | 0.023 | 0.157 | 0.001 | 0.001 | 0.005 |
| Inconel 740 | bal. | 32.03 | 21.74 | 0.370 | 0.220 | 0.676 | | 0.153 | 0.825 | 0.003 | 0.013 | |
| SINM | bal. | 22.16 | | 0.347 | 0.285 | 1.852 | | 0.081 | 35.185 | 0.001 | <0.001 | |
| Waspaloy | bal. | 25.84 | 16.19 | 3.234 | 0.531 | 1.036 | | | 0.232 | 0.003 | 0.008 | |

## References


[1] Miner, R. V., Gayda, J. & Maier, R. D. Fatigue and creep-fatigue deformation of several nickel-base superalloys at 650 °C. *Metall. Trans. A* **13**, 1755–1765 (1982).

[2] Bassini, E. *et al*. Study of the effects of aging treatment on Astroloy processed via hot isostatic pressing. *Materials* **12**, 1517 (2019).

[3] Lai, G. Y. An investigation of the thermal stability of a commercial Ni-Cr-Fe-Mo alloy (Hastelloy alloy X). *Metall. Trans. A* **9**, 827–833 (1978).

[4] Tawancy, H. M. Long-term ageing characteristics of Hastelloy alloy X. *J. Mater. Sci.* **18**, 2976–2986 (1983).

[5] Kim, D., Sah, I. & Jang, C. Effects of high temperature aging in an impure helium environment on low temperature embrittlement of Alloy 617 and Haynes 230. *J. Nucl. Mater.* **405**, 9–16 (2010).

[6] Kim, D., Sah, I. & Jang, C. Effects of aging in high temperature helium environments on room temperature tensile properties of nickel-base superalloys. *Mater. Sci. Eng. A* **528**, 1713–1720 (2011).

[7] Boehlert, C. J. & Longanbach, S. C. A comparison of the microstructure and creep behavior of cold rolled Haynes 230 alloy$^{TM}$ and Haynes 282 alloy$^{TM}$. *Mater. Sci. Eng. A* **528**, 4888–4898 (2011).

[8] Kaoumi, D. & Hrutkay, K. Tensile deformation behavior and microstructure evolution of Ni-based superalloy 617. *J. Nucl. Mater.* **454**, 265–273 (2014).

[9] The Japan Institute of Metals and Materials, editor. *Kinzoku data book, 4th rev ed* (Maruzen, 2004).

[10] Peng, T., Yang, B., Yang, G., Wang, L. & Gong, Z. Microstructural evolution and mechanical properties of Nimonic 105 alloy aged at 750 °C. *J. Alloy Compd.* **798**, 375–385 (2019).

[11] Shimanuki, Y. & Doi, H. Effect of aging treatment on microstructure and tensile properties of Udimet 520 and Ni-6.38%Al alloy. *Trans. JIM* **15**, 24–31 (1974).

[12] Xu, S., Dickson, J. I. & Koul, A. K. Grain growth and carbide precipitation in superalloy, Udimet 520. *Metall. Mater. Trans. A* **29**, 2687–2695 (1998).

[13] Harf, F. H. Effects of long-time elevated temperature exposures on hot-isostatically-pressed powder-metallurgy Udimet 700 alloys with reduced cobalt contents. *NASA Tech Memo* 83632 (1984).

[14] Devaux, A. *et al*. Mechanical properties and development of supersolvus heat treated new nickel base superalloy AD730. *MATEC Web Conf.* **14**, 01004 (2014).

[15] Sajjadi, S. A., Nategh, S. & Guthrie, R. I. L. Study of microstructure and mechanical

properties of high performance Ni-base superalloy GTD-111. *Mater. Sci. Eng. A* **325**, 484–489 (2002).

[16] Yang, C. *et al*. Improvement of stress-rupture life of GTD-111 by second solution heat treatment. *Mater. Des.* **45**, 308–315 (2013).

[17] Shingledecker, J. P. & Evans, N. D. Creep-rupture performance of 0.07C–23Cr–45Ni–6W–Ti, Nb austenitic alloy (HR6W) tubes. *Int. J. Pressure Vessels Pip.* **87**, 345–350 (2010).

[18] Tokairin, T. *et al*. Investigation on long-term creep rupture properties and microstructure stability of Fe–Ni based alloy Ni–23Cr–7W at 700 °C. *Mater. Sci. Eng. A* **565**, 285–291 (2013).

[19] Jonsta, Z., Jonsta, P., Konecna, K. & Gabcova, M. Phase analysis of nickel superalloy Inconel 738 LC. *Commun. Sci. Lett. Univ. Žilina* **12**, 90–94 (2010).

[20] Render, M., Santella, M. L., Chen, X., Tortorelli, P. F. & Cedro III, V. Long-term creep-rupture behavior of alloy Inconel 740/740H. *Metall. Mater. Trans. A* **52**, 2601–2612 (2021).

[21] Zhong, A. H., Gu, Y. F., Yuan, Y. & Shi, Z. A new wrought Ni–Fe-base superalloy for advanced ultra-supercritical power plant applications beyond 700 °C. *Mater. Lett.* **109**, 38–41 (2013).

[22] Merrick, H. F. & Floreen, S. The effects of microstructure on elevated temperature crack growth in nickel-base alloys. *Metall. Trans. A* **9**, 231–236 (1978).
